# Ultrasound guided technique for central venous catheters cannulation in critical care patients

**DOI:** 10.1186/2197-425X-3-S1-A71

**Published:** 2015-10-01

**Authors:** JA Soler, MD Casado, SM Botías, L Capilla, G Quintanilla, MC Lorente, DO Torres, A Burruezo, A Carrillo

**Affiliations:** Intensive Care Unit, Morales Meseguer Hospital, Murcia, Spain

## Introduction

Central venous catheters (CVC) can help with diagnosis and treatment of the critically ill. CVC cannulation risks arterial puncture and other complications and should be performed in as few attempts as possible. In the past, anatomical landmarks on the body surface were used to find the correct place to insert these catheters, but ultrasound imaging is now available.

## Objectives

To analyze safety and effectiveness of CVC cannulation by ultrasound guided (USG) technique in critical care setting.

## Methods

Prospective and observational study of all CVC cannulated in ICU patients, except those peripherally inserted, during 9 months in a university teaching hospital. Demographic and clinical data as well as variables related to cannulation were collected. Results are expressed as mean ± standard deviation and percentages. Comparisons between variables were performed by Student´s t-test and Pearson´s chi-squared test.

## Results

A total of 175 CVC were cannulated in 118 patients. On the first attempt, USG technique was chosen in 93 CVC (53.1%) being the successful procedure in 107 CVC (61.1%). There were no differences between USG and anatomical landmark technique regarding sex (women 35.9% vs. 38.5%; p = 0.727) or age (66.0 ± 14.3 years vs. 67.3 ± 14.2 years; p = 0.553). According to CVC indication, USG cannulation was chosen mainly for renal replacement therapy (22.6% vs. 9.8%; p = 0.023). Nonetheless, there were no differences regarding haemodinamic management (62.4% vs. 73.0%; p = 0.128), parenteral nutrition (8.6% vs. 3.7%; p = 0.179) and temporary pacemaker (5.4% vs. 11.0%; p = 0.173). USG technique was mainly performed by residents (57.8% vs. 33.3%; p = 0.011), in more severe patients (SOFA 9.0 ± 4.3 vs. 7.3 ± 4.3; p = 0.018), in patients with another CVC 30 days before (36.6% vs. 20.7%; p = 0.021) and if platelets transfusion was needed (10.8% vs. 2.4%; p = 0.030) without differences in local complications (29.0% vs. 32.9%; p = 0.578). In the same way, first attempt successful rate was higher in USG procedure (68.8% vs. 48.8%; p = 0.007) contrary to what happened with the need for technique change (3.2% vs. 20.7%; p < 0.001). Finally, there were no severe complications in both groups.

## Conclusions

In critical care setting, ultrasound guided CVC cannulation is a safe procedure which use is preferred in more severe patients. In the same way, USG technique is related to higher successful rate on the first attempt than conventional procedure.
